# Longer Telomere Length and its Association with Lower Levels of C-Peptide

**DOI:** 10.3389/fendo.2017.00244

**Published:** 2017-09-14

**Authors:** Min Yang, Ping Jiang, Chenghao Jin, Jinshan Wang

**Affiliations:** ^1^Department of Respiration, Tianjin First Center Hospital, Tianjin, China; ^2^Department of Transplantation, Tianjin First Center Hospital, Tianjin, China

**Keywords:** telomere, C-peptide, obesity, endocrinology, National Health and Nutrition Examination Survey

## Abstract

**Background:**

Telomeres undergo shortening with each cell division, which could be accelerated by increase obesity and is also related to endocrinology systems. In this study, we aimed to examine the complex association between telomere, C-peptide, and obesity as well as chronic inflammation in a large population-based cross-sectional survey.

**Methods:**

We used data from a community-based population study, where around 1,382 participants were recruited and had telomere length measured. The association of telomere length with C-peptide was studied using multiple linear regression models. We also examined if obesity, measured by body mass index (BMI), and inflammation could affect this observed association.

**Results:**

Around 48% of these participants were men and 52% were women. The average ages were 51.7 years old for men and 49.1 years old for women. After controlling for age and sex, 1 U increase of telomere length was associated with −0.17 (−0.28, −0.06) unit decrease of C-peptide. Additionally controlling for BMI, the association magnitude was decreased to −0.13 (−0.23, −0.04). Further adjusting for inflammation biomarker did not change the effect estimates.

**Conclusion:**

Longer telomere was associated with lower levels of C-peptide. This association could be attenuated by adjusting for obesity.

## Introduction

Telomeres are repeated deoxyribonucleic acid sequences and surrounding proteins at the end of chromosomes. The length of telomere (TL) shortens gradually overtime during each cell division and recognized as a promising biomarker of aging (1). TL declines with increasing age in human beings and has been associated with type 2 diabetes (2, 3), cardiovascular disease (4, 5), obesity (6), insulin resistance (4, 7), and other chronic conditions (5, 8, 9). However, its association with C-peptide has rarely been investigated (10–12). A case–control study found TL to be associated with C-peptide (10), similar results were observed in a cross-sectional study (13). However, other studies did not find a correlation between TL and C-peptide (11, 12). C-peptide is a short polypeptide with 31-amino-acid and relates insulin’s A-chain to its B-chain in the proinsulin molecule. In diabetes and other diseases, peripheral blood C-peptide assessment can be used to distinguish between certain diseases with similar clinical features, such as type 1 and type 2 diabetes. Examining the relationship of TL with C-peptide could provide additional information regarding the role of telomere in metabolic diseases regulation given the high prevalence of diabetes (14, 15). Because obesity was associated with both TL (6) and C-peptide (16), the association of TL and C-peptide could be confounded by obesity. In this study, we hypothesized that longer TL could be associated with lower levels of C-peptide, which could be attenuated by obesity. This hypothesis is to be evaluated by using the large community-based data in The National Health and Nutrition Examination Survey (NHANES).

## Materials and Methods

### Study Population

The NHANES survey is a continuous study that is being performed by the Centers for Disease Control and Prevention (CDC) that provides estimates of the health, chronic disease, and nutrition status of U.S. residents. In this study, we use data from the 1999 to 2000 cycles, where participants were given options to provide blood samples and had their telomere measured. The data collection of the NHANES has been approved by the National Center for Health Statistics Ethics Review Board. Written informed consent was acquired from each NHANES participant.

### Telomere Length Assessment

The details of telomere length assessment were described elsewhere (17). Processed DNA was provided by the laboratory of the NHANES Division in the CDC, U.S. Peripheral blood samples were used to extract the DNA, which were then stored at −80°C. The assessment of the TL was conducted in the laboratory in UCSF using the standard quantitative polymerase chain reaction method to estimate the relative telomere length by comparing it to the standard reference length of DNA (reported as T/S ratio). All samples were tested triple in three different days. We calculated the mean of the T/S ratio values and marked the measured values which were greater than 0.4 compared with the mean T/S ratio as an outlier. In total, around 1.3% of all samples contained outliers.

### C-Peptide Measurements

C-peptide was measured using the radioimmunoassay that is a competitive assay. A fixed and known amount of iodine 125-labeled C-peptide was added and incubated in this assay. During this incubation period, the radiolabeled C-peptide could bind to available sites. Then, the unbounded and bounded antibodies are separated by precipitation. The C-peptide levels are then estimated by plotting a calibration curve.

### Statistical Analysis

**Figure 1:**
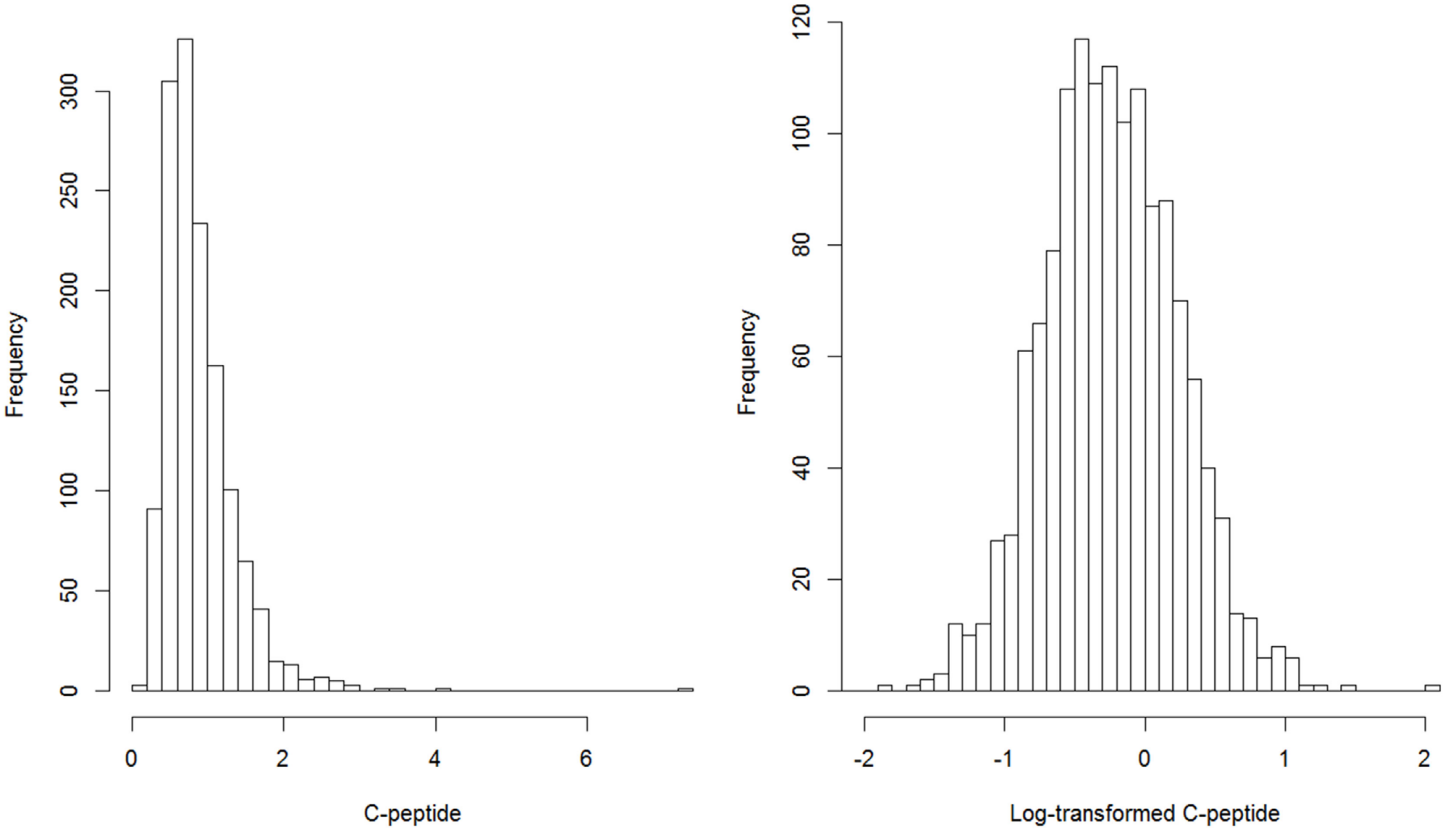
Distribution of C-peptide and Log-transformed C-peptide.

**Figure 2:**
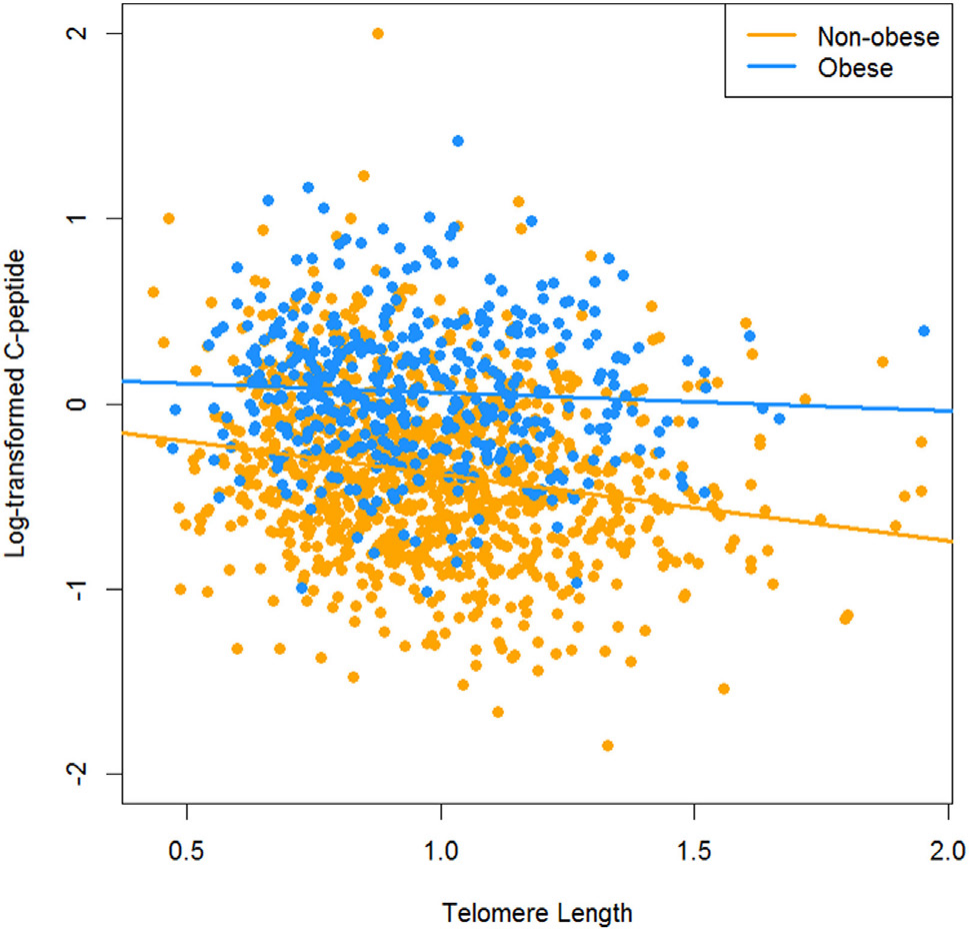
The relationship between C-peptide and telomere length by obesity.

We log-transformed C-peptide to make it normally distributed. The distributions of C-peptide and log-transformed C-peptide were presented in Figure 1. We used multiple linear regression models to assess and test the association between C-peptide and TL. Continuous variables were presented by mean and SDs and category variables were described as number and proportions. Four models were used to estimate the association. The first model was the crude analysis where the dependent variable is the log-transformed C-peptide and independent variable is telomere length. The second model was adjusted by age and sex. The third model was further adjusted for body mass index (BMI). The fourth model was additionally controlled by C-reactive protein (CRP). We also presented the scatter plot of telomere length and C-peptide by obesity (Figure 2). Sampling survey design was taken into account of in the multiple regression models using the *survey* package in R. Two sided *P*-value <0.05 was considered as statistically significant. All analyses were conducted using R 3.3.

## Results

Basic characteristics of study participants are presented by sex in Table 1. In total, 1,382 participants had both telomere and C-peptide data available, of which 665 (48.1%) were men and 717 (51.9%) were women. The average ages were 51.7 years old for men and 49.1 years old for women. Women had slightly longer TL compared with men (0.98 vs. 0.97 T/S ratio), but lower log-transformed C-peptide (−0.27 vs. −0.21 nmol/L).

**Table 1:** Basic characteristics of study participants.

Variables	Men (*n* = 665)	Women (*n* = 717)	All (*n* = 1,382)
Age (years)	51.7 (18.6)	49.0 (19.0)	50.3 (18.8)
Telomere length (T/S ratio)	0.97 (0.26)	0.98 (0.23)	0.97 (0.25)
Log-transformed C-peptide (nmol/L)	−0.21 (0.48)	−0.27 (0.49)	−0.24 (0.49)
C-reactive protein (mg/dL)	0.39 (0.82)	0.55 (0.72)	0.47 (0.77)
Height (cm)	173.4 (7.8)	160.0 (7.5)	166.6 (10.1)
Weight (kg)	84.0 (17.6)	72.1 (16.9)	77.8 (18.2)
Body mass index (kg/m^2^)	27.8 (5.0)	28.0 (6.1)	28.0 (5.6)

Figure 1 describes the distribution of original C-peptide and log-transformed C-peptide. After log-transformation, the distribution of C-peptide approaches normal distribution. Table 2 describes the association of TL with C-peptide in this population. We first categorized TL to thirds and used categorical TL as the independent variable to estimate its association with log-transformed C-peptide. Compared with the lowest thirds, people in the highest thirds of TL had −0.20 (95% CI: −0.26, −0.14) nmol/L lower C-peptide in the first model. The effect size was attenuated after adjusting for age and sex in the second model (β: marginal effect = −0.11, 95% CI: −0.18, −0.05). After further controlling for BMI, the magnitude was decreased to −0.09 (95% CI: −0.14, −0.03). In addition, adjustment of CRP did not change the association. We also modeled TL as a continuous variable, 1 U increase of TL was association with −0.13 (95% CI: −0.22, −0.04) decreased of log-transformed C-peptide when controlling for age, sex, BMI, and CRP in the fourth model. Analysis by obesity status was presented in Table 3; however, the interaction term between obesity and TL was not statistically significant.

**Table 2:** Association between telomere length and C-peptide, β (95% CI).

Telomere length	Model 1	Model 2	Model 3	Model 4
Thirds 1 (0.43−)	Reference	Reference	Reference	Reference
Thirds 2 (0.84−)	−0.09 (−0.16, −0.03)	−0.05 (−0.11, 0.01)	−0.03 (−0.08, 0.02)	−0.03 (−0.08, 0.02)
Thirds 3 (1.06−)	−0.20 (−0.26, −0.14)	−0.11 (−0.18, −0.05)	−0.09 (−0.14, −0.03)	−0.09 (−0.14, −0.03)
Continuous	−0.33 (−0.43, −0.23)	−0.18 (−0.29, −0.07)	−0.13 (−0.23, −0.04)	−0.13 (−0.22, −0.04)

*Results from Model 1 were the crude estimates. Model 2 was adjusted for age and sex, while Model 3 was further adjusted for body mass index, followed by Model 4 with C-reactive protein adjustment*.

**Table 3:** Association between telomere length and C-peptide by obesity, β (95% CI).

Telomere length	Obese	Non-obese
Quarters 1 (0.43−)	Reference	Reference
Quarters 2 (0.79−)	−0.001 (−0.10, 0.10)	−0.02 (−0.10, 0.06)
Quarters 3 (0.95−)	0.001 (−0.10, 0.11)	−0.12 (−0.21, −0.04)
Quarters 4 (1.13−)	−0.009 (−0.10, 0.12)	−0.12 (−0.20, −0.03)
Continuous	−0.001 (−0.16, 0.16)	−0.19 (−0.31, −0.07)

*Model was adjusted for age, sex, and C-reactive protein*.

## Discussion

In this study, we hypothesized TL to be associated with C-peptide and this association could be attenuated by obesity. We examined the association in a large population-based survey using the NHANES data and found that longer TL was association with lower levels of C-peptide. This observed association remained statistically significant after controlling for multiple covariates, but was attenuated with obesity adjustment. In addition, taking into account of CRP in the models did not change the association magnitude, suggesting that TL might influence C-peptide through biological pathways beyond inflammation.

Length of telomere has been shown to be predictive of diabetes and related glycemic traits in both observational population studies (2, 3, 7) and animal experiments (18, 19). However, its association with C-peptide has seldom been examined. A case–control study with 15 participants did not find a significant correlation between TL and C-peptide (11). Another study in 52 new infants of mothers with gestational diabetes and pre-gestational diabetes assessed both TL and C-peptide in this sample, but did not extensively examined their association (12). However, in a large study with 556 newly diagnosed diabetic patients, TL was negatively correlated with C-peptide although the association did not reach a significance level (13). Similar findings were observed in a case–control study of pancreatic cancer, in which TL was statistically significantly correlated with C-peptide in both cases and controls (10). Our results were in line with these findings, but with large sample size and more detailed investigation.

Previous studies have shown obesity and inflammation were associated with TL (6, 20–22). Obese people had shorter TL compared with their lean peers (6) and oxidative stress and inflammation could accelerate TL shortening (22–24). Likewise, obesity and inflammation are two important risk factors for glycemic traits including diabetes, insulin resistance, and C-peptide (16, 25, 26). It is thus nature to hypothesize obesity and inflammation could be the common causes of both TL and C-peptide. In this study, we tested these relationship hypothesis and the results did not contradict it. When controlling for BMI, the association magnitude was attenuated by approximately 30%. This means obesity could be able to accelerate TL shortening and to drive C-peptide levels up. However, when additionally controlling for CRP, the magnitude did not change any more. As both the obesity and inflammation were involved in metabolic disorder pathogenesis, additionally controlling for one of them when the other was already adjusted for would not affect the models too much.

There were several strengths associated with this study. First, telomere length was measured by a lab using well-established methods. Second, the study participants were randomly selected by NHANES. The sample size was large and representative of the wide age span, allowing the generalization of these results. Finally, we made adjustments in the statistical models for a number of covariates, including age, sex, BMI, and CRP. Findings indicated that the relationship between telomere length and C-peptide was independent of these factors. However, limitations should also be acknowledged. The nature of cross-sectional study makes it difficult to draw causal inference based on our analyses. Both BMI and CRP could also be affected by TL, and additional adjustment of BMI and CRP could underestimate the total effect of TL on C-peptide. However, in this study, all these biomarkers were measured at the same time, and it is difficult to distinguish the cause and consequence among them. Thus, both the unadjusted and adjusted estimates were provided. Although multiple regression models were used, there are still many other variables that might confound the observed association. Residual confounding should be taken into account of in future analyses. Besides that, this study did not include genetic variants or telomerase activity assessment. Examining these two biomarkers could definitely provide more evidence to justify our results. Additional limitations include the use of *P*-value as statistical significance criteria. We should acknowledge that the statistically significant finding in our study may not imply clinical significance in practice and the lack of statistical significant evidence does not mean the evidence of absence, e.g., the interaction between obesity and telomere. Thus, our findings should be interpreted with caution.

In summary, this study shows that longer TL was associated with C-peptide in the participants of NHANES, suggesting that TL may be regarded as a predictive biomarker of glycemic traits. Although our findings are derived in a cross-sectional survey, the finding of TL as a prognostic biological aging biomarker for C-peptide, independent of obesity and CRP, may increase our knowledge to assess disease risk and may also increase our understanding of the underlying pathways and mechanisms for the association of biological aging with a wide range of metabolic disorders.

## Ethics Statement

This study was carried out in accordance with the recommendations of National Center for Health Statistics Ethics Review Board with written informed consent from all subjects. All subjects gave written informed consent in accordance with the Declaration of Helsinki. The protocol was approved by the National Center for Health Statistics Ethics Review Board.

## Author Contributions

JW and MY designed and analyzed the data. MY, PJ, and CJ wrote the manuscript. All the authors critically revised and approved the manuscript.

## Conflict of Interest Statement

The authors declare that the research was conducted in the absence of any commercial or financial relationships that could be construed as a potential conflict of interest.
